# Factors for Patient Trust and Acceptance of Medical Artificial Intelligence

**DOI:** 10.1001/jamanetworkopen.2026.0815

**Published:** 2026-03-05

**Authors:** Ana Bracic, Kayte Spector-Bagdady, Sophie Towle, Rina Zhang, Cornelius A. James, W. Nicholson Price

**Affiliations:** 1 Department of Political Science, Michigan State University, East Lansing; 2 Michigan Bioethics, University of Michigan Medical School, Ann Arbor; 3 University of Michigan Law School, Ann Arbor; 4 Department of Learning Health Sciences, University of Michigan Medical School, Ann Arbor; 5 Department of Internal Medicine, University of Michigan Medical School, Ann Arbor; 6 Department of Pediatrics, University of Michigan Medical School, Ann Arbor; 7 Centre for Advanced Studies in Bioscience Innovation Law, University of Copenhagen Faculty of Law, Copenhagen, Denmark; 8 Department of Obstetrics and Gynecology, University of Michigan Medical School, Ann Arbor

## Abstract

**Importance:**

Artificial intelligence (AI) is increasingly used in clinical care, but widespread adoption requires patient trust. Trust may be enhanced through systemic governance mechanisms or frontline clinicians providing a human in the loop for AI oversight. However, it is unclear how different approaches specifically influence patient trust in the use of medical AI.

**Objective:**

To determine the extent to which patient trust in and choice of medical scenarios involving AI are associated with governance mechanisms, clinician presence, performance, and data quality.

**Design, Setting, and Participants:**

This preregistered conjoint survey study was conducted online among a diverse national sample of English-speaking US adults with access to the internet between December 11, 2024, and January 1, 2025. Respondents were presented with hypothetical AI-assisted diagnosis scenarios and paired visits featuring 6 purely randomized attributes: the presence of a clinician, AI performance (relative to general practitioners and specialists), governance (US Food and Drug Administration approval, Mayo Clinic certification, local hospital certification), and AI data quality. Respondents chose their preferred visit, provided up to a single-sentence open-ended response explaining their choice, and then rated their trust in the diagnosis they would receive in each of the 2 visit choices presented to them. Respondents repeated the exercise 6 times, evaluating 12 hypothetical visits in total, yielding 36 000 observations (12 per respondent).

**Main Outcomes and Measures:**

The primary outcomes were patient choice of a hypothetical medical encounter and patient trust in that encounter, measured on a 1 (would not trust at all) to 5 (would trust a great deal) response scale. Average marginal component effects (AMCEs) were estimated using linear regression. Qualitative responses were coded to elucidate reasoning.

**Results:**

A total of 3000 participants completed the survey (1644 [54.8%] women; mean [SD] age, 48 [16] years), including 382 Black respondents (12.7%), 504 Hispanic respondents (16.8%), and 1855 White respondents (61.9%), with most respondents having some college or more (1989 respondents [66.3%]), and 1270 respondents (42.4%) having income between $50 000 and $99 000. The factor associated with the largest change in likelihood of patient choice was AI performance; performance at or above the specialist level was associated with increasing the probability of selecting a visit by 24.8% (95% CI, 23.4%-26.2%; *P* < .00025) and 32.5% (95% CI, 31.0%-33.9%; *P* < .00025), respectively. The presence of a clinician was associated with increasing the probability of selecting a visit by 18.4% (95% CI, 17.3%-19.5%; *P* < .00025). Respondents who received information on representative AI training data also were more likely to prefer that visit scenario. Respondents preferred all forms of AI governance compared with none. Qualitative responses emphasized AI performance and clinician presence as primary factors in choice of visit.

**Conclusions and Relevance:**

In this survey study of patient trust in and choice of medical AI, AI performance, clinician presence, disclosure of representative data, and systemic governance were associated with increased respondent trust in and preference for clinical encounters. These findings suggest that ensuring resource-appropriate combinations of these tools is an important step in helping AI achieve its transformative potential for the health system.

## Key Points

**Question:**

What factors are associated with patient trust in and choice of medical artificial intelligence (AI)?

**Findings:**

In this survey study of 3000 US adults, respondents were significantly more likely to trust in and choose medical AI in scenarios with better AI performance, US Food and Drug Administration approval, national certification, local certification, the presence of a clinician, and the use of representative data.

**Meaning:**

These findings suggest that adopters of medical AI should consider implementing oversight mechanisms to increase patient trust and acceptance of its use.

## Teaser

This survey study examines the associations of patient trust in and choice of medical scenarios involving artificial intelligence with receiving information on governance mechanisms, clinician presence, performance, and data quality.

## Introduction

Artificial intelligence (AI) is increasingly transforming the practice of medicine across a wide range of clinical tasks. These include predicting readmission rate or mortality, monitoring patients for the onset of sepsis, analyzing radiographic images, generating clinical encounter summaries or discharge notes, screening for cancer, and answering medical questions.^1,2^ AI can also bring clinical interventions to patients who otherwise lack access. For example, one AI-powered, technician-operated tool is cleared by the US Food and Drug Administration (FDA) to autonomously diagnose diabetic retinopathy, as opposed to merely advising an ophthalmologist or optometrist, whom patients may have difficulty accessing. AI has the potential to broaden care to underserved patients, particularly by increasing access to general and specialized health care in rural communities, urban communities, and lower-resource settings.

Realizing the potential of AI will require progress on several fronts, including curation of accurate and diverse data to train systems, rigorous development of those systems, adoption of AI by hospitals or other clinical environments, clinician training, workflow integration, and governance.^3,4^ Adoption of many front-line AI systems will also be substantially influenced by whether and how much patients trust and prefer the technologies^5^ and the clinicians and health organizations using them.^6^ Patient trust has been linked to a number of health behaviors and subjective outcomes,^7^ improved patient satisfaction, and engagement in shared decision-making.^8^ Demonstrated competence can also build trust in hospital systems,^9^ but recent studies have found a low baseline of patient trust in health care systems to use AI responsibly and protect patients from harm.^6^ Of course, distrust may be warranted; medical AI systems can suffer from bias, poor performance, and inadequate governance.^3,4^ AI systems that patients prefer and trust are more likely to be adopted by hospitals and health systems and chosen by patients (assuming they have a choice).^10,11^ However, patients who distrust AI may eschew newly available AI-based care or even avoid current care when AI becomes integrated.

Patient trust in medical AI likely both parallels and departs from our understanding of trust in physicians. In both, patients likely cannot independently evaluate medical performance. Patient trust in clinicians accordingly correlates with perceptions of empathy, communication, and knowledge of the patient, rather than directly with competence or trustworthiness.^12^ Factors driving patient trust in AI are still being elucidated.

Prior research has considered whether patients are more likely to trust or accept AI systems vs human clinicians and for which tasks.^13^ Lee and Rich^14^ found associations between mistrust of medicine generally and mistrust of medical AI, focusing on the experience of Black patients. Frank et al^10^ found that patients are more likely to prefer an AI diagnosis to a human diagnosis when they trust AI and when they believe clinicians will not consider patients’ unique characteristics. Robertson et al^15^ found that respondents were substantially more likely to select the use of AI when it was demonstrated to be accurate or a primary care clinician nudged them to use it.

In this study, we assess 2 commonly discussed approaches in implementing medical AI that could increase patient trust: (1) systemic governance mechanisms to ensure and communicate that AI systems are safe and effective and (2) frontline clinicians to provide oversight of individual AI decisions by serving as a human in the loop of the AI system.^16^ We also consider the impact of disclosed training data quality and AI performance. In this study, we ask how those approaches may influence patient trust in and choice of the use of medical AI in their care. Understanding the impacts of these approaches can shape how developers, policymakers, and health systems implement governance and oversight strategies.

### Systemic Governance

There are 3 prominent loci for governance of medical AI: federal, national nonfederal, and local health systems. Federal regulation comes principally via review by the FDA, leading to market authorization. FDA approval or authorization is a significant factor in trust of many medical technologies.^17^ Many, but not all, medical AI systems fall under the FDA’s jurisdiction as medical devices.^18^ The FDA has authorized marketing of more than 1000 AI medical devices.^19^

Key stakeholders, including the Coalition for Health AI and the FDA, have proposed an additional national-level governance mechanism to complement FDA regulation: national assurance laboratories or assurance providers operated by academic medical centers (AMCs) or similar entities.^20^ AI developers could bring their AI to these laboratories for assessment of technical performance, expected uses and features, and bias.

Local governance can address AI performance variation across different care environments.^4^ Some hospitals and health systems, especially higher-resourced AMCs, already have robust local governance structures.^2^ Unfortunately, local governance requires resources that are limited in many care environments.

Instead of asking which governance mechanisms best ensure implementation of safe and effective medical AI,^4^ which is outside the scope of this study, we ask a separate question: how these mechanisms are associated with patient choice and trust. Government regulations, certifications, standards, and guidelines may impact the perceived integrity and trustworthiness of medical AI.^21,22^ But all these levels of governance require resources in a resource-constrained world. It remains an open empirical question which mechanisms impact patients deciding whether to trust medical AI used in their care.

### Humans in the Loop

A second dual-purpose mechanism that might enhance patient choice and trust in conjunction with safety and effectiveness is the presence of a clinician who can be a human in the loop. In this mechanism, the clinician operates in concert with an AI system, evaluating, implementing, modifying, and/or rejecting a recommendation from AI. Humans in the loop are a common safety and effectiveness intervention for AI systems across many domains, including medicine.^4^

The presence of humans in the loop may also enhance patient trust in medical AI by trying to ensure safety and effectiveness.^23^ To the extent that clinicians mediate the patient experience of medical AI, clinician presence may shape patients’ trust of AI, or, conversely, the use of low-quality AI might decrease patient trust in the clinician.^24^

Finally, we consider 2 underlying characteristics of AI systems that may be linked to trust: (1) AI system performance, compared with a clinician’s, and (2) use of a representative training dataset, as nonrepresentative data can lead to biased AI and poorer performance for some patients.^24,25,26^ We recognize that these attributes, as well as systemic governance, are often opaque to patients; to the extent that they influence patient trust and choice, they may warrant disclosure as part of AI transparency in patient care.

System governance mechanisms, humans in the loop, improved performance, and representative training data each could enhance patient trust and choice (as well as quality of care), but designing effective systems requires understanding how much each of these factors matter to the patient and what characteristics are important. Empirical evaluation of patient preferences can advance that understanding.

## Methods

This survey study was determined exempt from review by the Michigan Medicine institutional review board. Informed consent was obtained from all respondents. This study is reported following the American Association for Public Opinion Research (AAPOR) reporting guideline (eTable 1 in Supplement 1) and the Discrete Choice Experiment Reporting Checklist (DIRECT) (eTable 2 in Supplement 1).

Our preregistered conjoint survey study of 3000 respondents was fielded to a diverse national sample by Verasight between December 11, 2024, and January 1, 2025, and preregistered on December 1, 2024, at AsPredicted. Verasight recruited panelists using a combination of probability and nonprobability methods. Verasight assigned each eligible panelist a probability of being selected based on the most recent population benchmarks from the American Community Survey (October 2024). Demographic data are based on self-identification by respondents and were provided by Verasight. Race and ethnicity were categorized as Black, Hispanic, White, or other and were assessed because health care preferences may vary by race or ethnicity.^24,25,26^

A multistage choice-based conjoint analysis allowed us to test how attributes related to data quality, performance, governance, and clinician oversight were associated with patient choices regarding medical AI. This approach has been widely used to elicit patient preferences across various contexts, including cancer treatment^27^ and primary care delivery.^28^ It quantifies the relative importance of different attributes and captures trade-offs patients make in health care choices, which is appropriate in the resource-constrained context of medical AI.

Respondents were presented with a hypothetical scenario in which they visit a medical facility about a rash (eMethods in Supplement 1). The scenario involved taking a photograph of the rash, which was then analyzed by a medical AI model that provided an initial diagnosis of the rash. We focused on a single stage of a relatively straightforward, broadly applicable diagnostic case with moderate risk to minimize the presence of complicating factors and to keep the task simple for respondents. This task nevertheless implicates each of our attributes. There is inherent challenge in differentiating possible diagnoses (dry skin, psoriasis, scabies). There is also evidence of diagnostic accuracy disparities across skin tones, making both physician judgment and representativeness of training data potentially critically important to diagnostic accuracy.^29^ Governance mechanisms are implicated in overseeing these issues.

Respondents were then presented serially with several pairs of hypothetical visits. They were informed that all involved the use of medical AI and that all cost the same. For each pair of visits, respondents were first asked to choose which visit they would prefer, to explain their choice in a sentence or less, and to rate how much they would trust each diagnosis on a scale from 1 (would not trust at all) to 5 (would trust a great deal).

Table 1 shows a pair of hypothetical visits as they would have appeared to a respondent. The visits feature 6 randomized attributes, listed in Table 2. The order of the attributes was randomized for each pair presented. We considered 4 factors: (1) the presence of a clinician, standing in for oversight via a human in the loop; (2) AI performance relative to general practitioners and specialists; (3) AI governance at various levels, represented by FDA approval (federal), Mayo Clinic certification (national nonfederal, standing in for national assurance laboratories by using a well-respected brand of medical excellence), or local-hospital certification; and (4) information on AI data quality (eg, training on representative or nonrepresentative data,^26^ or no training data information provided).

**Table 1.** Example Pair of Hypothetical Visits^a^

Attribute	Hypothetical visit 1	Hypothetical visit 2
Information received on AI data quality	AI was trained on a disproportionately white, male, and wealthy dataset	No information received
AI performance	Better than a specialist	About the same as a general practitioner
Clinician present during the visit	Yes	No
Is AI FDA approved?	No	Yes
Is AI certified by the local hospital?	Yes	No
Is AI certified by the Mayo clinic?	No	No

Abbreviations: AI, artificial intelligence; FDA, Food and Drug Administration.

^a^ Participants were prompted with the following text: “Please consider the following hypothetical scenario. You have a rash that you’re concerned about. You’ve done some googling and you think it could be one of 3 things: dry skin, psoriasis, or scabies. You decide to go to a medical facility near you to have the rash examined. When you enter, a medical professional directs you to a booth that takes a photograph of your rash. The photograph is then analyzed by a medical artificial intelligence (AI) model that provides an initial diagnosis of your rash. For the next few minutes, we are going to ask you questions about this scenario. We will describe several pairs of hypothetical visits to this medical facility. All of the visits involve the use of medical AI and all cost the same. For each pair of visits, please indicate which one you would prefer to experience. Even if you aren’t entirely sure, please indicate which of the two you would prefer to experience. We will also ask you a few questions about each hypothetical visit.”

**Table 2.** Profile Attributes and Attribute Levels

Attribute	Levels
Information received on AI data quality	No information received
	AI was trained on a disproportionately white, male, and wealthy dataset
	AI was trained on a representative United States population dataset
AI performance	Better than a specialist
	About the same as a specialist
	About the same as a general practitioner
	Worse than a general practitioner
Clinician present during the visit	Yes
	No
Is AI FDA approved?	Yes
	No
Is AI certified by the local hospital?	Yes
	No
Is AI certified by the Mayo clinic?	Yes
	No

Abbreviations: AI, artificial intelligence; FDA, Food and Drug Administration.

Each respondent repeated the exercise 6 times, evaluating 12 hypothetical visits in total, yielding 36 000 total observations. Presentation of attribute levels was randomized. Randomization checks confirmed proper level balance (eTable 3 in Supplement 1).

### Statistical Analysis

Our a priori power calculation was for visit choice and specified 6 tasks per respondent, at most 4 attribute levels, and an effect size of 0.05 for a single attribute–level comparison. We conducted this power analysis for a half sample to ensure sufficient power for our preregistered gender subset analysis. A sample of 1500 is powered at 99%, with Type S error of 0% and type M error of 1.04.

Statistical analyses were conducted in Stata version 15.1 (StataCorp) and R version 4.3.1 (R Project for Statistical Computing), using linear regression with standard errors clustered on the respondent to derive the average marginal component effect (AMCE) of the attributes on both patient choice (binary) and trust (ordinal) outcome variables.^30^ This presents the association of each attribute, averaging across all other attributes, but does not offer insight into any unique combination of attributes. Associations from the conjoint analysis were considered significant at the Bonferroni-corrected *P* < .00025 level. Statistical significance of differences between conjoint experiment coefficients was determined using clustered 2-tailed Wald tests, and significance was set at *P* < .05. Two authors (S.T. & R.Z.) coded the responses to the open-ended question (eMethods and eTable 4 in Supplement 1); we report concepts mentioned by at least 100 respondents. Paired 2-tailed *t* tests were used to examine differences in means for concept mentions.

## Results

The final sample included 3000 English-speaking US adults with internet access (1644 [54.8%] women; 1334 men [44.5%]; 22 [0.7%] individuals selecting other) who provided high-quality survey responses. The mean (SD) age was 48 (16) years. There were 1011 respondents (33.7%) with at most a high school education, 858 respondents (28.6%) with some college or a 2-year degree, and 1131 respondents (37.7%) with a 4-year or postgraduate degree. There were 988 respondents (33.0%) who earned less than $50 000 per year, 1270 respondents (42.4%) who earned between $50 000 and $99 999 per year, 452 respondents (15.1%) who earned between $100 000 and $149 999 per year, and 288 respondents (9.6%) who earned more than $150 000 per year. In terms of race and ethnicity, 382 respondents (12.7%) were Black, 504 respondents (16.8%) were Hispanic, 1855 respondents (61.9%) were White, and 258 respondents (8.6%) selected other race or ethnicity (eTable 5 in Supplement 1).

**Figure 1.**
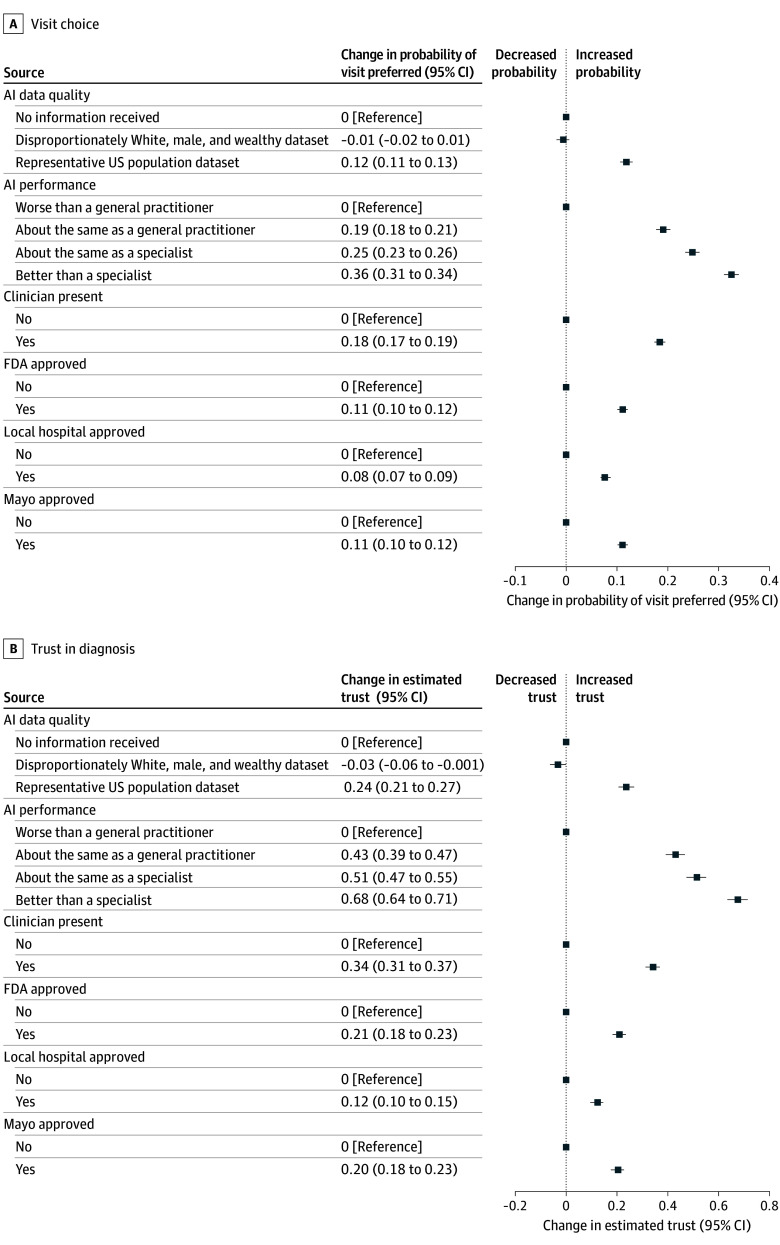
Forest Plots of Attribute Associations With Visit Choice and Trust in Diagnosis This figure plots the change associated with each attribute in visit choice and trust outcomes. A, Probability that a patient would choose a hypothetical visit (in a binary choice where 1 indicates the visit was preferred) based on the different attribute levels. B, Association with expected trust, on a scale from 1 (would not trust at all) to 5 (would trust a great deal). For each attribute, multiple levels are possible. The data points show the change from baseline (eg, no clinician present) for other attribute levels (eg, clinician present). All changes are statistically significantly different from baseline at *P* < .00025, except changes in choice or trust based on a biased training dataset (ie, disproportionately White, wealthy, and male), which were not statistically significantly different from baseline. AI indicates artificial intelligence; FDA, US Food and Drug Administration.

Figure 1A plots AMCEs with 95% CIs to show the association of our attributes with the probability of selecting a hypothetical visit. Figure 1B similarly shows attribute associations with expected respondent trust in the diagnosis received in the visit. eTable 6 and eTable 7 in Supplement 1 report regression results corresponding to Figure 1.

As Figure 1 shows, the associations with respondent trust closely paralleled those with respondent choice. For ease of reading, we report only AMCEs for visit choice; each represents the change in probability of preferring a visit based on an attribute level, relative to baseline. AMCEs for respondent trust are reported in eTable 7 and the eAppendix in Supplement 1. All reported associations from the conjoint analysis are significant at the Bonferroni-corrected *P* < .00025 level unless otherwise noted.

### Clinician Oversight and AI Performance

Respondents showed a clear preference for a human in the loop. The AMCE was 0.184 (95% CI, 0.173-0.195), meaning that respondents were 18.4% (95% CI, 17.3%-19.5%) more likely to choose a visit with a clinician than one with no clinician (baseline). Information on AI performance was associated with respondent choice as much as or more than clinician oversight. The greatest increase in preference was observed for AI performance at or above specialist level (AMCE, 0.248 [95% CI, 0.234 to 0.262] and AMCE, 0.325 [95% CI, 0.310 to 0.339], respectively). Above-specialist performance was nearly 3 times as important as FDA approval (AMCE, 0.111 [95% CI, 0.101 to 0.121]). The association for medical AI that performs as well as a general practitioner (AMCE, 0.191 [95% CI, 0.177 to 0.205]) was nearly the same as the association for clinician oversight (AMCE, 0.184 [95% CI, 0.173 to 0.195]; difference in coefficients for a clustered 2-tailed Wald test, 0.007 [95% CI, −0.011 to 0.025]; *P* = .46), although the information on medical AI with a performance equivalent to a general practitioner was associated with a greater increase in trust than clinician oversight and was the only instance where trust and patient choice notably differed. All Wald tests are reported in eTables 8 to 17 in Supplement 1.

### Data Quality

Respondents preferred AI trained on a representative US population dataset to AI about which they received no training data information (AMCE, 0.119; 95% CI, 0.106 to 0.131). We also included the possibility of AI trained on a disproportionately White, male, and wealthy population because it reflects the datasets currently frequently used in practice and raises concerns about specific biases.^26^ Respondents neither favored nor disfavored AI trained on these data compared with AI about which they received no training data information.

### Governance

Respondents preferred all forms of AI governance compared with no governance. They preferred FDA-approved medical AI to unapproved AI, medical AI certified by the Mayo Clinic to uncertified AI, and medical AI certified by a local hospital to uncertified AI. The increases in preference associated with FDA approval (AMCE, 0.111 [95% CI, 0.101 to 0.121]) and Mayo Clinic certification (AMCE, 0.111 [95% CI, 0.101 to 0.121]) were the same size (difference, 0 [95% CI, −0.014 to 0.014]; *P* = .96). The increase for local hospital certification (AMCE, 0.078 [95% CI, 0.068 to 0.088]) was smaller (difference between local and FDA, 0.033 [95% CI, 0.019 to 0.047]; *P* < .001; difference between local and Mayo Clinic, 0.034 [95% CI, 0.020 to 0.048]; *P* < .001).

Results from patient choice and trust models were robust to controlling for respondent characteristics and weighting (eTable 18, eTable 19, and eFigure 1 in Supplement 1). Women and men showed no significant differences in their preferences based on AI quality, performance, governance, or clinician oversight, but women exhibited an overall lower level of trust in visits involving AI generally (eTable 20, eTable 21, eFigure 2 and eFigure 3 in Supplement 1).

**Figure 2.**
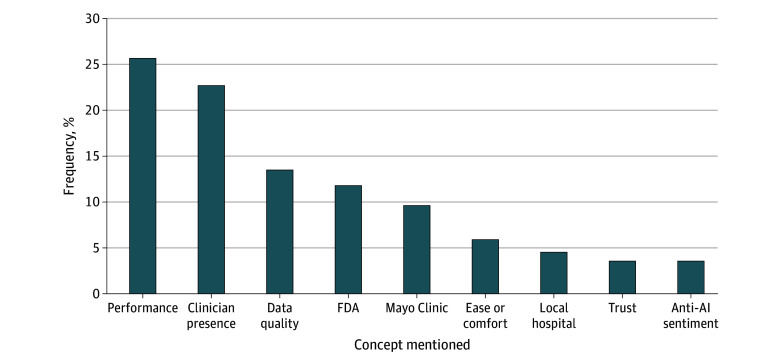
Bar Graph of Frequency of Concepts Mentioned in Open-Ended Responses This figure shows the frequency with which respondents mention a particular concept responding to the first choice in the conjoint experiment. Concepts mentioned by more than 100 respondents are included. Differences between ease or comfort vs local hospital and trust vs anti–artificial intelligence (AI) sentiment are not significant. All other differences are significant at the *P* < .05 level. FDA indicates US Food and Drug Administration.

In their explanations for their choice of visit, respondents were most likely to mention AI performance (25.7%) and clinician presence (22.67%) (Figure 2). Mentions of concepts roughly tracked the conjoint results. Figure 2 shows the frequency with which respondents mentioned a particular concept responding to the first choice in the conjoint experiment. Concepts mentioned by more than 100 respondents are included. The following differences were significant at the *P* < .05 level: AI performance vs clinician presence (difference, 3.0% [95% CI, 0.6%-5.4%]), clinician presence vs data quality (difference, 9.2% [95% CI, 7.2%-11.2%]), FDA vs Mayo Clinic (difference, 2.2% [95% CI, 0.7%-3.7%]), Mayo Clinic vs ease or comfort (difference, 3.9% [95% CI, 2.6%-5.3%]), and local hospital vs trust (difference, 1.0% [95% CI, 0.0%-2.0%]). Differences between data quality vs FDA, ease or comfort vs local hospital, and trust vs anti-AI sentiment were not significant (eTables 22-29 in Supplement 1).

## Discussion

The findings of this survey study indicate that when the information was available and salient, patient trust in and choice of medical AI encounters were associated with representative data, clinician presence, and the existence of federal, national nonfederal, or local governance—but that AI performance was the most significant factor associated with shaping patient choice and trust. Our results provide novel contributions to the literature in 3 ways. First, we demonstrated attribute association with patient choice, rather than relying on explicit statements that attributes matter. Second, we identified comparative importance of different attributes, which is especially relevant in a reality of constrained resources. Third, we evaluated the importance of different levels of governance mechanisms, an underexplored area in relation to patient trust and choice.

Notably, local hospital validation was associated with influencing trust and choice less than either FDA approval or national-level validation via the Mayo Clinic. All forms of validation are potentially important for ensuring that AI is safe and effective: FDA approval ensures a baseline level of functioning, national nonfederal review (eg, assurance laboratories) can help ensure clinical applicability, and local review can demonstrate that an AI system actually works well in situ.^4^ The local level may be most practically relevant to individual patients but is the governance level most vulnerable to resource disparities and other differences between local health systems. The lack of nonfederal influence on patient trust suggests a need for increased resource investment to strengthen local validation, since it is nondelegable for the foreseeable future, given differences across care environments.^18^

Meaningfully, clinician presence was associated with a greater change in patient trust and choice than any individual form of governance. However, effective clinician governance presents substantial challenges: not only are clinicians limited in their ability effectively to oversee medical AI performance, but the availability of adequately trained clinicians is limited, especially in low-resource settings, for underserved populations, and in underserved specialties.^21^ Patient preference for clinicians is unsurprising, given the importance of the clinician-patient relationship. Nevertheless, strong patient preferences for a clinician in the loop limit the potential of medical AI to expand health services availability to lower-resourced settings.

Thus, it is critically important to recognize that the most important factor associated with patient choice was neither any form of governance nor the presence of a clinician—it was medical AI system performance. AI performing at the level of a generalist had as large an association with patient trust and choice as the presence of a clinician (and larger than any form of governance). AI performance at or above the level of a specialist had, respectively, the greatest and second-greatest association with patients’ preference for and trust in a particular medical encounter. To be sure, while independent for study design reasons in this study, these issues are not independent in clinical settings. High AI performance is ensured and certified at a basic level by national-level organizations, such as the FDA and assurance laboratories, and at the contextual level by local health systems.^4^ Furthermore, clinicians have the potential—although a challenging potential to realize—to ensure AI performance at the individual patient level. Patient trust and acceptance involve all of these elements.

In our study, these attributes were transparent and highly salient to trust and choice, but in clinical situations, information about governance, data, and performance are rarely conveyed to patients—even information about how clinicians interact with AI may be unclear. Increasing transparency regarding these attributes may increase patient trust and consent to incorporating AI in their care.

Further research could consider combinations of these attributes and the implications of inevitable resource-based tradeoffs to inform decisions about policy investment and system design. While patients would likely prefer expert clinicians, familiar with AI, with ample time to oversee medical AI systems, that combination is costly and likely unavailable in all but the most resource-rich environments—if at all.

### Limitations

Our study has several limitations. Our results assume that patients know both that AI is involved in their care and the presence or absence of various attributes. It remains an open question how much patients will be informed about the use of AI (including whether informed consent demands such information^25,31^), whether patients will be given other information about the AI systems involved in their care (eg, through the use of model facts labels for AI^32^), and patient’s trust in that information. Our hypothetical scenario considered one specific medical condition with relatively moderate risk (diagnosis of a rash with a straightforward differential), and the factors impacting patient trust and choice likely vary across different conditions of differing risk. Our scenario was also simplified to minimize cognitive load and accordingly could not capture subtle and complex nuances of an actual clinical scenario, which also likely influence patient trust and choice. Our conjoint design does not allow evaluations of combinations of governance (for instance, how much more likely are patients to choose an encounter if the AI is FDA approved, Mayo Clinic certified, and local-hospital certified). Our sample includes English-speaking adults with internet access who agreed to participate in the online panel and answered a survey in December and January. Additionally, preferences expressed in online survey experiments might not match real-world behaviors.

## Conclusions

In this survey study of patient trust in and choice of medical AI, AI performance, clinician presence, disclosure of representative data, and systemic governance were associated with increased respondent trust in and preference for medical encounters with AI. These findings suggest that ensuring resource-appropriate combinations of these tools is an important step in helping AI achieve its transformative potential for health, as it is increasingly integrated into medical practice, and helps increase the reach of care to underserved populations.

## Notes

Supplement 1. eMethods. eTable 1. American Association for Public Opinion Research (AAPOR) reporting guidelines checklist eTable 2. Reporting Checklist for Discrete Choice Experiments in Health (DIRECT) (Ride et al. 2024) eTable 3. Frequencies of values presentation for each attribute in the conjoint experiment. eTable 4. Intercoder reliability for open-ended responses eTable 5. Summary statistics of the population covariates eTable 6. Average marginal component effects (AMCE) for patient choice, full sample eTable 7. Average marginal component effects (AMCE) for patient trust, full sample eTable 8. Clustered two-tailed Wald test for a difference between the AMCE coefficients for AI performance (about the same as a general practitioner) and Clinician present, patient choice eTable 9. Clustered two-tailed Wald test for a difference between the AMCE coefficients for FDA approved and Mayo approved, patient choice eTable 10. Clustered two-tailed Wald test for a difference between the AMCE coefficients for FDA approved and Local hospital approved, patient choice eTable 11. Clustered two-tailed Wald test for a difference between the AMCE coefficients for Mayo approved and local hospital approved, patient choice eTable 12. Clustered two-tailed Wald test for a difference between the AMCE coefficients for AI performance (about the same as a general practitioner) and AI performance (about the same as a specialist), patient choice eTable 13. Clustered two-tailed Wald test for a difference between the AMCE coefficients for AI performance (about the same as a specialist) and AI performance (better than a specialist), patient choice eTable 14. Clustered two-tailed Wald test for a difference between the AMCE coefficients for AI performance (about the same as a general practitioner) and Clinician present, patient trust eTable 15. Clustered two-tailed Wald test for a difference between the AMCE coefficients for FDA approved and Mayo approved, patient trust eTable 16. Clustered two-tailed Wald test for a difference between the AMCE coefficients for FDA approved and Local hospital approved, patient trust eTable 17. Clustered two-tailed Wald test for a difference between the AMCE coefficients for Mayo approved and local hospital approved, patient trust eTable 18. Average marginal component effects (AMCE) for patient choice, full sample with demographic controls eTable 19. Average marginal component effects (AMCE) for patient trust, full sample with demographic controls eTable 20. Average marginal component effects (AMCE) for patient choice, women eTable 21. Average marginal component effects (AMCE) for patient choice, men eTable 22. Paired two-tailed t-test for a difference in means between mentions of AI performance and mentions of a clinician eTable 23. Paired two-tailed t-test for a difference in means between mentions of a clinician and mentions of AI data quality eTable 24. Paired two-tailed t-test for a difference in means between mentions of AI data quality and mentions of the FDA. eTable 25. Paired two-tailed t-test for a difference in means between mentions of the FDA and mentions of the Mayo Clinic. eTable 26. Paired two-tailed t-test for a difference in means between mentions of the Mayo Clinic and mentions of ease or comfort eTable 27. Paired two-tailed t-test for a difference in means between mentions of the Mayo Clinic and mentions of ease or comfort eTable 28. Paired two-tailed t-test for a difference in means between mentions of local hospital and mentions of trust eTable 29. Paired two-tailed t-test for a difference in means between mentions of trust and expressions of anti-AI sentiment eFigure 1. Patient choice AMCEs, weighted full sample with 95% confidence intervals eFigure 2. Patient choice AMCEs, women subset with 95% confidence intervals eFigure 3. Patient choice AMCEs, men subset with 95% confidence intervals eAppendix.

Supplement 2. Data Sharing Statement
