# Supplementary material for: Factors for Patient Trust and Acceptance of Medical Artificial Intelligence
Source: JAMA Netw Open. 2026 Mar 5;9(3):e260815. doi: 10.1001/jamanetworkopen.2026.0815 (PMC12964161; doi:10.1001/jamanetworkopen.2026.0815)
Supplement: Supplement 1. — eMethods. eTable 1. American Association for Public Opinion Research (AAPOR) reporting guidelines checklist eTable 2. Reporting Checklist for Discrete Choice Experiments in Health (DIRECT) (Ride et al. 2024) eTable 3. Frequencies of values presentation for each attribute in the conjoint experiment. eTable 4. Intercoder reliability for open-ended responses eTable 5. Summary statistics of the population covariates eTable 6. Average marginal component effects (AMCE) for patient choice, full sample eTable 7. Average marginal component effects (AMCE) for patient trust, full sample eTable 8. Clustered two-tailed Wald test for a difference between the AMCE coefficients for AI performance (about the same as a general practitioner) and Clinician present, patient choice eTable 9. Clustered two-tailed Wald test for a difference between the AMCE coefficients for FDA approved and Mayo approved, patient choice eTable 10. Clustered two-tailed Wald test for a difference between the AMCE coefficients for FDA approved and Local hospital approved, patient choice eTable 11. Clustered two-tailed Wald test for a difference between the AMCE coefficients for Mayo approved and local hospital approved, patient choice eTable 12. Clustered two-tailed Wald test for a difference between the AMCE coefficients for AI performance (about the same as a general practitioner) and AI performance (about the same as a specialist), patient choice eTable 13. Clustered two-tailed Wald test for a difference between the AMCE coefficients for AI performance (about the same as a specialist) and AI performance (better than a specialist), patient choice eTable 14. Clustered two-tailed Wald test for a difference between the AMCE coefficients for AI performance (about the same as a general practitioner) and Clinician present, patient trust eTable 15. Clustered two-tailed Wald test for a difference between the AMCE coefficients for FDA approved and Mayo approved, patient trust eTable 16. Clustered two-tailed Wald tes [file jamanetwopen-e260815-s001.pdf]

Supplemental Online Content

Bracic A, Spector-Bagdady K, Towle S, Zhang R, James CA, Price WN. Factors for patient trust and acceptance of medical artificial intelligence. *JAMA Netw Open*. 2026;9(3):e260815. doi:10.1001/jamanetworkopen.2026.0815

eMethods.
eTable 1. American Association for Public Opinion Research (AAPOR) reporting guidelines checklist
eTable 2. Reporting Checklist for Discrete Choice Experiments in Health (DIRECT) (Ride et al. 2024)
eTable 3. Frequencies of values presentation for each attribute in the conjoint experiment.
eTable 4. Intercoder reliability for open-ended responses
eTable 5. Summary statistics of the population covariates
eTable 6. Average marginal component effects (AMCE) for patient choice, full sample
eTable 7. Average marginal component effects (AMCE) for patient trust, full sample
eTable 8. Clustered two-tailed Wald test for a difference between the AMCE coefficients for AI performance (about the same as a general practitioner) and Clinician present, patient choice
eTable 9. Clustered two-tailed Wald test for a difference between the AMCE coefficients for FDA approved and Mayo approved, patient choice
eTable 10. Clustered two-tailed Wald test for a difference between the AMCE coefficients for FDA approved and Local hospital approved, patient choice
eTable 11. Clustered two-tailed Wald test for a difference between the AMCE coefficients for Mayo approved and local hospital approved, patient choice
eTable 12. Clustered two-tailed Wald test for a difference between the AMCE coefficients for AI performance (about the same as a general practitioner) and AI performance (about the same as a specialist), patient choice
eTable 13. Clustered two-tailed Wald test for a difference between the AMCE coefficients for AI performance (about the same as a specialist) and AI performance (better than a specialist), patient choice
eTable 14. Clustered two-tailed Wald test for a difference between the AMCE coefficients for AI performance (about the same as a general practitioner) and Clinician present, patient trust
eTable 15. Clustered two-tailed Wald test for a difference between the AMCE coefficients for FDA approved and Mayo approved, patient trust
eTable 16. Clustered two-tailed Wald test for a difference between the AMCE coefficients for FDA approved and Local hospital approved, patient trust
eTable 17. Clustered two-tailed Wald test for a difference between the AMCE coefficients for Mayo approved and local hospital approved, patient trust

eTable 18. Average marginal component effects (AMCE) for patient choice, full sample with demographic controls
eTable 19. Average marginal component effects (AMCE) for patient trust, full sample with demographic controls
eTable 20. Average marginal component effects (AMCE) for patient choice, women
eTable 21. Average marginal component effects (AMCE) for patient choice, men
eTable 22. Paired two-tailed t-test for a difference in means between mentions of AI performance and mentions of a clinician
eTable 23. Paired two-tailed t-test for a difference in means between mentions of a clinician and mentions of AI data quality
eTable 24. Paired two-tailed t-test for a difference in means between mentions of AI data quality and mentions of the FDA.
eTable 25. Paired two-tailed t-test for a difference in means between mentions of the FDA and mentions of the Mayo Clinic.
eTable 26. Paired two-tailed t-test for a difference in means between mentions of the Mayo Clinic and mentions of ease or comfort
eTable 27. Paired two-tailed t-test for a difference in means between mentions of the Mayo Clinic and mentions of ease or comfort
eTable 28. Paired two-tailed t-test for a difference in means between mentions of local hospital and mentions of trust
eTable 29. Paired two-tailed t-test for a difference in means between mentions of trust and expressions of anti-AI sentiment
eFigure 1. Patient choice AMCEs, weighted full sample with 95% confidence intervals
eFigure 2. Patient choice AMCEs, women subset with 95% confidence intervals
eFigure 3. Patient choice AMCEs, men subset with 95% confidence intervals
eAppendix.

This supplemental material has been provided by the authors to give readers additional information about their work.

eMethods

1 Ethical Research Practices (Consent)

UNIVERSITY OF MICHIGAN
CONSENT TO BE PART OF A RESEARCH STUDY

NAME OF STUDY AND RESEARCHERS
Trust, Governance, and Humans in the Loop in Clinical Artificial Intelligence (AI)
Principal Investigator: Kayte Spector-Bagdady, JD, MBE, University of Michigan Medical School
Co- Principal Investigator: Nicholson Price, PhD, JD, University of Michigan Law School
Co-Investigator: Cornelius James, MD, MHPE, University of Michigan
Co-Investigator: Ana Bracic, PhD, Michigan State University

GENERAL INFORMATION
Topic: We're doing a study to learn more about patient preferences regarding the use of AI in their care. If you choose to participate, we will ask you to read hypothetical medical scenarios and answer questions about your preferences related to AI. For this study, we would like survey about 3,000 people via online survey. We expect the survey to take about 15 minutes.

YOUR PARTICIPATION IN THIS STUDY IS VOLUNTARY Participating in the survey is voluntary. You don't have to participate if you'd rather not. You can skip any questions that you don't want to answer, whatever the reason, and you don't have to tell us why.

WHAT RISKS MIGHT RESULT FROM BEING IN THIS STUDY? It's possible that some of the questions may make you feel uncomfortable. If a question makes you uncomfortable, you can skip to the next question.

HOW WILL WE PROTECT YOUR INFORMATION? Surveys will be conducted via Qualtrics, a secure online survey platform. Survey data will be de-identified and labeled with a code, rather than your name or other details someone could use to identify you. We plan to publish what we learn from this study, but we won't include any personal information that could reveal who participated. Your collected de-identified information may be shared with other researchers who are part of this study team.

HOW COULD YOU BENEFIT FROM THIS STUDY? Participating in the study won't benefit you directly. We hope what we learn will help improve AI practices in the future.

HOW WILL WE COMPENSATE YOU FOR BEING PART OF THE STUDY? To thank you for taking part in our study you will receive an incentive through Versasight which will be points

equivalent to $1 after you have completed the survey.

CONTACT INFORMATION
To find out more about the study, to ask a question or express a concern about the study, or to talk about any problems you may have as a study subject, you may contact one of the following:

Study Coordinator: Jessie Milne
Email: jmmilne@med.umich.edu
Mailing Address:
2800 Plymouth Rd.
NCRC Building 14, G016
Ann Arbor, MI 48109

Principal Investigator: Kayte Spector-Bagdady
Email: kaytesb@med.umich.edu
Telephone: 734-764-9886
Mailing Address:
2800 Plymouth Rd.
NCRC Building 14, G016
Ann Arbor, MI 48109

You may also express a concern about a study by contacting the Institutional Review Board:

University of Michigan Medical School Institutional Review Board (IRBMED)
2800 Plymouth Road
Building 520, Room 3214
Ann Arbor, MI 48109-2800
734-763-4768
E-mail: irbmed@umich.edu

If you are concerned about a possible violation of your privacy or concerned about a study, you may contact the University of Michigan Health System Compliance Help Line at 1-866-990-0111.

By providing information to the researcher(s), I am agreeing to participate in this research:

- ○ I agree to participate in this research
- ○ I do NOT agree to participate in this research

2 Pre-registration

The conjoint experiment was pre-registered before data collection began at AsPredicted (#201939). We pre-registered gender subset analyses in addition to the full sample analyses. An anonymized pdf of the pre-registration can be found at https://aspredicted.org/nhgp-g2tg.pdf. The pre-registration also lists the additional OLS analyses that are presented in this Appendix (see Tables).

3 Case Prompt and a Sample Choice Set

In the first part of the survey we will ask you some questions about medical artificial intelligence (AI). Medical AI is the use of AI tools to analyze medical data to help improve patient care.

These questions will include some terms. We'll define the terms first.

------------page break------------

AI performance: Medical AI can be used to diagnose some medical conditions. After the AI examines thousands of cases, its performance is checked by comparing its diagnoses to those made by doctors. Some AI systems can be as accurate as medical experts, while others may not be as reliable.

------------page break------------

AI data quality: In order to recognize patterns and draw conclusions, AI has to be trained on existing data. Ultimately, the quality of AI's predictions is only as good as the quality of its training data. Sometimes, the data are representative of the general population. Sometimes, they are not.

------------page break------------

FDA approval: The US Food and Drug Administration (FDA) is a government agency. The FDA ensures that medical devices, including some AI, are safe, effective, and properly manufactured before they can be marketed and used in healthcare.

------------page break------------

The Mayo Clinic: The Mayo Clinic is a medical center renowned for its comprehensive healthcare services and cutting-edge research. It is one of the world's most respected hospitals.

------------page break------------

Thank you for reading these descriptions.

Now, please consider the following hypothetical scenario.

------------page break------------

You have a rash that you're concerned about. You've done some googling and you think it could be one of 3 things: dry skin, psoriasis, or scabies. You decide to go to a medical facility near you to have the rash examined.

When you enter, a medical professional directs you to a booth that takes a photograph of your rash. The photograph is then analyzed by a medical artificial intelligence (AI) model that provides an initial diagnosis of your rash.

For the next few minutes, we are going to ask you questions about this scenario. We will describe several pairs of hypothetical visits to this medical facility for this issue. All of the visits involve the use of medical AI and all cost the same.

------------page break------------

These hypothetical visits refer to the scenario you just read about the rash. Of these two hypothetical visits, please indicate which one you would prefer to experience. Even if you aren't entirely sure, please indicate which of the two you would prefer to experience.

	Hypothetical visit 1	Hypothetical visit 2
Clinician present during the visit	Yes	No
AI performance	Better than specialist	About the same as a general practitioner
Is AI FDA approved?	No	Yes
Is AI certified by the Mayo clinic?	No	No
Is AI certified by the local hospital?	Yes	No
Information received on AI data quality	AI was trained on a disproportionately white, male, and wealthy data set	No information received

4 Outcome Measures

The following questions served as the dependent variables in our models:

1. Patient Choice: "Which of these hypothetical visits would you prefer to experience?" Hypothetical visit 1/Hypothetical visit 2, select one

2. Patient Choice (open-ended): "Why would you prefer this visit? Please answer in a sentence or less."

3. Trust: How much would you trust a diagnosis if you received it during each visit? Please indicate your answer on the scale from "would not trust at all" to "would trust a great deal"

5 Coding of Open-Ended Responses

Two of the coauthors coded 3000 statements (responding to the first of six conjoint pairs) using the codebook below. After a third co-author performed the intercoder reliability analysis in R, the two coding coauthors worked together to reconcile any discrepancies in the codes. Table A1 below shows Krippendorff's Alphas and indicates a high degree of agreement on the reported codes.

The following instructions were given to the individual coding the open-ended responses to the "Why would you prefer this visit? Please answer in a sentence or less." question.

The coders were instructed to read respondents' answers strictly, rather than interpreting what they thought respondents might mean.

MIDAS AI Study 1 Conjoint Experiment Codebook

Coding rules for open-ended answers to:

"Why would you prefer this visit? Please answer in one sentence or less."

Don't know
0 - respondent does not indicate that they don't know
1 - respondent indicates they don't know

No reason/no difference

0 - respondent doesn't say that there's no reason for their decision or that there is no difference
1 - respondent says that there's no reason for their decision or that there is no difference

Example:
"Just picked no difference in my opinion"
"None"
"No reason"

Gibberish
0 - you can tell what the respondent is trying to say
1 - you can't tell what the respondent is trying to say

Example:
"Jsjsjndjs"
"Fluff you'll hghjk Idk hello full"

Had to choose
0 - respondent does not mention that the choice was compulsory
1 - respondent mentions that the choice was compulsory

1 example:
"Because I had to choose"

Clinician
0 - respondent does not mention a clinician
1 - respondent mentions a clinician

NOTE: Respondents were told either that there was a clinician present during the visit or that there wasn't a clinician present.

AI data quality
0 - respondent does not mention the training data, sample, or representativeness/population
1 - respondent mentions the training data, sample, or representativeness/population

NOTE: respondents were randomly exposed to the following:
- "No information received"
- "AI is trained on disproportionately white, male, and wealthy dataset"
- "AI is trained on a representative US population dataset"

AI performance
0 - respondent does not mention AI performance
1 - respondent mentions AI performance

NOTE: respondents were randomly exposed to:
- “About the same as a general practitioner”
- “Worse than a general practitioner”
- “About the same as a specialist”
- “Better than a specialist”

FDA
0 - respondent does not mention the FDA
1 - respondent mentions the FDA

NOTE: Respondents were told that the AI was either FDA approved or not.

Mayo
0 - respondent does not mention the Mayo clinic
1 - respondent mentions the Mayo clinic

NOTE: Respondents were told that the AI was either approved by the Mayo clinic or not.

Local hospital
0 - respondent does not mention the local hospital
1 - respondent mentions the local hospital

NOTE: Respondents were told that the AI was either approved by the local hospital or not.

Neither
0 - respondent does not mention that they would choose neither visit
1 - respondent mentions that they would choose neither visit

Both
0 - respondent does not mention that they would choose both visits
1 - respondent mentions that they would choose both visits

Anti AI

0 - respondent does not express anti-AI sentiment
1 - respondent expresses anti-AI sentiment

Accuracy/precision/credibility/reliability → Substantive quality
0 - respondent does not mention accuracy/precision
1 - respondent mentions accuracy/precision

"Has more credibility compared to visit 2"
"Seems more reliable"
"An AI is better at comparing images than a doctor."

Trust
0 - respondent does not mention trust
1 - respondent mentions trust

Security/privacy
0 - respondent does not mention security or privacy
1 - respondent mentions security or privacy
*access to data

"Because it seems to be very secure"
"Because it's the one more safe and accommodate"
"This one sounds less dangerous"

Health risk/safety
0 - respondent does not mention risk and safety
1 - respondent mentions risk and safety
*medical outcomes

"Because it's the one more safe and accommodate"
"This one sounds less dangerous"

Ease/convenience
0 - respondent does not mention that their choice seemed better
1 - respondent mentions that their choice seemed better

"It would be easier"
"More convenient"

Comfort
0 - respondent does not mention comfort
1 - respondent mentions comfort

“Feel more comfortable”
“I feel comfortable”
“More assuring”
“It fits what I am looking for”
“It feels more my style”

Better (nebulous)
0 - respondent does not mention that their choice seemed better
1 - respondent mentions that their choice seemed better

“More logical”
“makes sense”

IF YOU ARE NOT SURE

- Leave the row blank and highlight it so we can discuss
- General rule is to be conservative. If you’re not sure what the respondent meant, code it as gibberish.

eTables

eTable1: American Association for Public Opinion Research (AAPOR) reporting guidelines checklist

Checklist Item	Response
Data collection strategy	The research team developed and pretested a survey instrument containing a conjoint experiment. The pretest consisted of cognitive interviews with men and women in their 20s, 30s, 40s, 60s, and 70s; the pretest population includes students, professionals, and retirees, and is racially diverse. The survey instrument was programmed in Qualtrics. We passed the Qualtrics QSF file to Verasight, which then administered the survey.
Who sponsored the research and who conducted it	University of Michigan sponsored the research. Additional funding was provided to researchers by the NIH and the Greenwall Foundation (KSB) and the Novo Nordisk Foundation (WNP). Research was conducted by University of Michigan and Michigan State University researchers.
Measurement tools/instruments	Survey instrument containing a conjoint survey experiment. See the instrument in eMethods (Case Prompt, Choice Set, and Outcome Measures).
Population under study	The population under study are English-speaking US adults with access to the internet.
Method used to generate and recruit the sample	The sample is drawn from a Verasight pre-recruited panel. Verasight uses a multi-mode panel recruitment strategy that leverages non-probability data with probability-based sampling (random address-based (ABS) and random text messaging). By combining multiple recruitment approaches, Verasight aims to maximize population coverage, appeal to different types of Americans who are more likely to use or respond to surveys in certain mediums and customize outreach to maximize response rates.<br><br>The final sample consisted of 3000 United States adults (age 18+) who provided active consent and passed all data quality assurance checks. People not covered by the design are individuals who do not have access to the internet, individuals who were excluded due to seasonal bias, and individuals who do not speak English.<br><br>Verasight uses sampling algorithms to select random, representative samples from the overall population of

	Verasight panelists. Verasight panelists can take surveys via email invitation or by checking their account via the web.<br><br>Verasight reported a survey response rate of 6.3%, without more specific breakdown. Verasight also reported that 437 respondents provided partial responses (we did not receive survey responses from those respondents).<br><br>Verasight respondents were awarded reward points for completing this survey. The dollar conversion for the reward points is $1.25.
Methods and modes of data collection	The survey was administered online.
Dates of data collection	Verasight piloted the survey to 133 respondents and fielded it to a total of 3000 respondents between December 11, 2024 and January 1, 2025.
Sample sizes	The final sample included 3000 English-speaking US adults with access to the internet.
How the data were weighted	Main analyses are unweighted. A robustness check reported in SI uses weights provided by Verasight. Using those weights, the data are weighted to match the October 2024 Current Population Survey on age, race/ethnicity, sex, income, education, region, and metropolitan status, as well as to a running three-year average of partisanship distributions from the Pew Research Center NPORS benchmarking surveys, and population benchmarks of 2020 presidential vote. The margin of sampling error, which accounts for the design effect and is calculated using the classical random sampling formula, is +/- 1.8%.
How the data were processed and procedures to ensure data quality	Verasight processed the data and delivered 3000 complete responses. There were therefore no missing data. The data quality assurance checks include confirming that all responses correspond with U.S. IP addresses, confirming no duplicate respondents, verifying the absence of non-human responses, removing any respondents who failed in-survey attention and/or straight-lining checks, and removing respondents that completed the survey in less than 30% of the median completion time.
A general statement acknowledging limitations of the design and data collection	The blended nature of panel recruitment includes non-probability sampling, which means that particular subgroups could be overrepresented in our sample. The sample also excludes people who do not have access to the internet and people who don't speak English. Other potential sources of

	error are nonresponse bias and measurement error due to potential misinterpretation of conjoint attributes. More generally, all research has limitations and we therefore acknowledge other unmeasured errors associated with all forms of survey research.

See for the AAPOR checklist, see https://aapor.org/standards-and-ethics/disclosure-standards/#1667933142550-55785157-2071

eTable 2: Reporting Checklist for Discrete Choice Experiments in Health (DIRECT) (Ride et al. 2024)

Section<br>Item	Response
**Purpose and rationale**	
1 Describe the real-world context and decision-maker that the hypothetical choice context seeks to replicate or inform	The real-world context is a patient seeking medical care in which artificial intelligence may be involved, and the hypothetical choice context seeks to replicate the decisions of that patient.
2 Provide a rationale for using a DCE to answer the research question	A DCE helps us quantify the relative importance of different attributes and capture trade-offs patients make in healthcare choices, which is appropriate in the resource-constrained context of medical AI.
**Attributes and levels**	
3 Describe how attributes and levels were derived (e.g. literature review, interviews, focus groups, expert input)	The attributes and levels were derived from literature review and expert input.
4 Provide the final list of attributes and levels	*AI data quality* (No information received; Disproportionately white, male, and wealthy dataset; Representative US population dataset)<br><br>*AI performance* (Worse than a general practitioner; About the same as general practitioner; About the same as specialist; Better than specialist)<br><br>*Clinician present* (No, Yes)

	FDA approved (No, Yes)<br><br>*Local hospital approved* (No, Yes)<br><br>*Mayo approved* (No, Yes)
**Experimental design**	
5 Report the number of alternatives per choice set and whether they were labelled or unlabeled	Each choice task presented 2 unlabeled alternatives (Hypothetical Visit 1 and Hypothetical Visit 2). This information is presented in Table 1.
6 Describe response options (e.g. forced choice, opt-out, status quo)	(1) Forced choice<br>[Hypothetical Visit 1/Hypothetical Visit 2]<br>(2) Open-ended response<br>[Why would you prefer this visit? Please answer in a sentence or less.]<br>(3) Likert scale, 1-5<br>[How much would you trust a diagnosis if you received it during each visit? Please indicate your answer on the scale from "would not trust at all" (1) to "would trust a great deal" (5)]
7 Describe the type of experimental design (e.g. orthogonal, D-efficient, Bayesian efficient, partial profile)	Each task presented a pair of profiles with all six attributes. Levels for all six attributes were randomly assigned for each profile.
8 Describe which effects are identified in the design (e.g. main effects, higher order interactions, functional form)	Average Marginal Component Effects for the full sample and for the gender subset analysis, both of which we pre-registered on December 1, 2024, at AsPredicted (#201939).
9 Describe the number of choice sets, blocks and choice sets per block	All respondents completed 6 tasks. There was no blocking.
10 Indicate how the experimental design was obtained (software, catalogue, other)	The experimental design was adapted from Programming Choice Experiments in Qualtrics by Matthew H. Graham (November 22, 2020.)
**Survey design**	
11 Provide a sample choice set and the instructions and background information given to respondents (e.g. providing the survey as an appendix)	Respondents first read the case prompt. They were presented with choice sets immediately after. The case prompt and a sample choice set are provided in eMethods.

12 Report any randomisation (e.g. choice set order, attribute order, alternative order, framing effects)	The order of the attributes was randomized for each pair presented. Attribute levels were randomly assigned.
13 Describe what was checked in piloting (e.g. understanding, respondent burden, timing, wording)	The pretest checked for understanding, timing, question wording and clarity, coverage of answer options, and respondent burden. The pretest consisted of cognitive interviews with men and women in their 20s, 30s, 40s, 60s, and 70s; the pretest population included students, professionals, and retirees, and was racially diverse.
14 Report whether information from the pilot was used to update the experimental design (e.g. priors, functional form of attributes) or survey design	Information from the first round of pretest led to the inclusion of the case prompt before the experiment. Minor wording changes corrected typographical errors. Feedback from elderly participants resulted in increasing the font size for better readability.
**Sample and data collection**	
15 Report respondent inclusion/exclusion criteria	The sampling criteria for this survey were:<br>1. Adults (age 18+)<br><br>The selection criteria for the final sample were:<br>1. Provided active consent<br>2. Passed all data quality assurance checks
16 Describe how data were collected (e.g. mail, personal interview, web survey)	Online survey. Participation took about 15 minutes.
17 Report the response rate or cooperation rate, if possible	Verasight reported a survey response rate of 6.3%, without more specific breakdown. Verasight also reported that 437 respondents provided partial responses (we did not receive survey responses from those respondents).
18 Report the final sample size and how the sample size was determined	The final sample size was 3000 English-speaking U.S. adults with access to internet. The sample size was determined with an ***a priori*** power analysis using a Shiny app for conjoint analysis power calculations developed on the basis of

	Stefanelli A, Lukac M (2020) Subjects, Trials, and Levels: Statistical Power in Conjoint Experiments. doi: 10.31235/osf.io/spkcy.<br><br>We first performed a calculation for a sufficiently powered gender subset. The gender subset analysis was a pre-registered analysis that required us to split the full sample approximately in half. We specified 6 tasks, at most 4 attributes, and a 0.05 effect size. We chose this effect size on the basis of Stefanelli and Lukac (2020) who identify it as a median effect size in their review of conjoint studies. A sample of 1500 is powered at 99%, has a Type-S error (the probability that the estimated coefficient has an incorrect sign) of 0% and the type-M error (exaggeration rate of the true effect) of 1.04.<br><br>We doubled a sufficiently powered gender subset to a full sample of 3000. With six tasks, at most four attribute values, and a 0.03 effect size, the full sample is powered at 97%. It has a Type-S error of 0% and a type-M error of 1.04.<br><br>We also conducted a retrospective power analysis for the white men subgroup. With N = 846, 6 tasks, at most 4 attributes, and a 0.05 effect size, this subsample is powered at 95% and has a Type-S error of 0% and a type-M error of 1.14.<br><br>The app is available at (https://mblukac.shinyapps.io/conjoints-power-shiny/).
19 Describe respondent characteristics and representativeness of target population, if known	Respondent demographics are presented in eTable 5.<br><br>The sample is a nationally diverse sample of English-speaking adults with access to

	Internet. The sample lacks seasonal representativeness, language representativeness (survey was not conducted in Spanish), and technological representativeness (respondents had access to internet).
**Econometric analysis**	
20 Indicate coding of data (e.g. effects, dummy, continuous) including definitions	*Patient choice, dummy* (Hypothetical visit 1, Hypothetical visit 2)<br>*Trust, categorical* (1 [would not trust at all], 2, 3, 4, 5 [would trust a great deal])<br>*AI data quality, categorical* (No information received [baseline]; Disproportionately white, male, and wealthy dataset; Representative US population dataset)<br>*AI performance, categorical* (Worse than a general practitioner [baseline]; About the same as general practitioner; About the same as specialist; Better than specialist)<br>*Clinician present, dummy* (No [baseline], Yes)<br>*FDA approved, dummy* (No [baseline], Yes)<br>*Local hospital approved, dummy* (No [baseline], Yes)<br>*Mayo approved, dummy* (No [baseline], Yes)<br><br>Demographic controls used for robustness checks:<br>*Age, categorical* (18-25, 26-35, 36-45, 46-55, 56-65, 66+)<br>*Education, categorical* (HS or less, Some college/2-yr degree, 4-yr/post-graduate degree)<br>*Income, categorical* (< $50,000, $50,000-$99,999, $100,000 - $149,999, > $150,000)<br>*Sex, categorical* (Male, Female, Other)<br>*Race, categorical* (White, Black, Hispanic Other)
21 Report whether any respondents were removed and why (e.g. suspected fraudulent responses, rationality tests)	Verasight removed respondents who did not pass data quality assurance checks.<br><br>The data quality assurance checks include confirming that all responses correspond

	with U.S. IP addresses, confirming no duplicate respondents, verifying the absence of non-human responses, removing any respondents who failed in-survey attention and/or straight-lining checks, and removing respondents that completed the survey in less than 30% of the median completion time.<br><br>Since we only received the final dataset of 3000 completes from Verasight, we do not have the information on how many respondents were removed.
22 Provide the rationale for model choice (e.g. conditional logit, mixed logit, latent class) and assumptions (e.g. error variance)	We use a simple linear regression estimator of the ACMEs (Average Marginal Component Effects). We use this method because it's appropriate for designs with completely independent randomization (Bansak et al. 2021). This method can be applied to outcome variables that are binary, rankings, and ratings without modification (Bansak et al. 2021). We therefore use it in models with both dependent variables, patient choice (binary) and trust (ordinal).
23 Report model specification	$choice_{ij} = \beta_0 + \beta_1 AI\ data\ quality_{ij} + \beta_2 AI\ performance_{ij} + \beta_3 Clinician_{ij} + \beta_4 FDA_{ij} + \beta_5 Mayo_{ij} + \beta_6 Local\ hospital_{ij} + \varepsilon_{ij}$<br><br>Dependent variable: binary indicator equal to 1 if respondent *i* chose profile *j* and 0 otherwise<br>Standard errors were clustered at the respondent level. Baseline categories are defined in section 20 of this table.<br><br>$trust_{ij} = \beta_0 + \beta_1 AI\ data\ quality_{ij} + \beta_2 AI\ performance_{ij} + \beta_3 Clinician_{ij} + \beta_4 FDA_{ij} + \beta_5 Mayo_{ij} + \beta_6 Local\ hospital_{ij} + \varepsilon_{ij}$<br><br>Dependent variable: trust rating for profile *j* by respondent *i*

	Standard errors were clustered at the respondent level. Baseline categories are defined in section 20 of this table.
**Reporting of results**	
24 Report the model performance, goodness of fit (if comparing models)	For the choice model (see section 23 of this table):<br>Multiple R-squared: 0.1362<br>Adjusted R-squared: 0.136<br><br>For the trust model (see section 23 of this table):<br>Multiple R-squared: 0.08775<br>Adjusted R-squared: 0.08752
25 Describe methods used for analysis of model results (e.g. calculation of marginal rate of substitution, attribute relative importance, welfare gain)	We calculated the Average Marginal Component Effects (AMCEs) for the full sample and for the gender subsets (Bansak et al. 2021).
26 Report measures of precision for the output(s) of interest (e.g. confidence intervals) and how these were derived	We started with 95% confidence intervals (and a corresponding p < 0.05). We then adjusted the p-value using a Bonferroni correction for multiple hypothesis testing. The number of tests includes all the attribute-level combinations, gender subsets, weighted and non-weighted models, and balancing checks, resulting in the corrected p-value of 0.00025 (0.05/197).

For more on the DIRECT checklist, see Ride J, Goranitis I, Meng Y, LaBond C, Lancsar E. A Reporting Checklist for Discrete Choice Experiments in Health: The DIRECT Checklist. Pharmacoeconomics. 2024 Oct;42(10):1161-1175. doi: 10.1007/s40273-024-01431-6.

eTable 3: Frequencies of values presentation for each attribute in the conjoint experiment

Attribute	Value	Frequency	Percent
AI data quality			
	No information received	11,946	33.2
	Disproportionately white, male, and wealthy dataset	12,060	33.5
	Representative US population dataset	11,994	33.3
AI performance			
	Worse than a general practitioner	8,958	24.9
	About the same as general practitioner	8,935	24.8
	About the same as specialist	9,037	25.1
	Better than specialist	9,070	25.2
Clinician present			
	No	17,953	49.9
	Yes	18,047	50.1
FDA approved			
	No	17,966	49.9
	Yes	18,034	50.1
Local hospital approved			
	No	17,964	49.9
	Yes	18,036	50.1
Mayo approved			
	No	18,168	50.5
	Yes	17,832	49.5

eTable 3: Frequencies of values presentation for each attribute in the conjoint experiment. Each attribute level was presented approximately equally across all choice tasks, with the largest deviation from expected frequency at 0.4%.

eTable 4: Intercoder reliability for open-ended responses

Variable	Krippendorff's Alpha
Don't know	0.931
No difference	0.792
Gibberish	0.465
Had to choose	0.329
**Clinician**	**0.96**
**AI data quality**	**0.921**
**AI performance**	**0.952**
**FDA**	**0.976**
**Mayo**	**0.994**
**Local hospital**	**0.969**
Neither	0.803
Both	-0.000334
**Anti-AI sentiment**	**0.821**
Accuracy/precision/credibility/reliability	0.49
**Trust**	**0.9**
Better	0.598
Security/privacy	0.691
Health risk/safety	0.573
**Ease/convenience**	**0.867**
Comfort	0.446
Certified (unspecified)	0.875

eTable 4: Intercoder reliability for open-ended responses to "Why would you prefer this visit? Please answer in a sentence or less." Krippendorff's Alpha scores for the codes reported in the manuscript are in bold.

eTable 5: Summary statistics of the population covariates

Population covariate	Category	N	percent
Age			
	18-25	257	8.57
	26-35	518	17.27
	36-45	599	19.97
	46-55	552	18.40
	56-65	503	16.77
	66+	571	19.03
	total	3000	100.00
Gender			
	female	1644	54.80
	male	1334	44.47
	other	22	0.73
	total	3000	100.00
Race			
	Black	382	12.74
	Hispanic	504	16.81
	Other	258	8.60
	White	1855	61.85
	total	2999	100.00
Income			
	less than $50,000	988	32.96
	$50,000 - $99,999	1270	42.36
	$100,000 - $149,999	452	15.08
	more than $150,000	288	9.61
	total	2998	100.00
Education			
	High school or less	1011	33.70
	Some college/2-year degree	858	28.60
	4-year/post-graduate degree	1131	37.70
	total	3000	100

eTable 6: Average Marginal Component Effects (AMCE) for Patient Choice, full sample.

Attribute	Estimate	Std. Err	Pr(>|z|)	
AI data quality (white, male, wealthy)	-0.006	0.006	0.311	
AI data quality (representative)	0.118	0.006	0.000	*
AI performance (as general practitioner)	0.191	0.007	0.000	*
AI performance (as specialist)	0.248	0.007	0.000	*
AI performance (better than specialist)	0.325	0.007	0.000	*
Clinician present	0.184	0.005	0.000	*
FDA approved	0.111	0.005	0.000	*
Local hospital approved	0.078	0.005	0.000	*
Mayo approved	0.111	0.005	0.000	*

Number of Obs. = 36000
Number of Respondents = 3000
'*' 0.00025

eTable 6: Average Marginal Component Effects (AMCE) for Patient Choice, full sample. This table corresponds to Figure 2 (Panel a) in the main manuscript. We apply a Bonferroni corrected p-value of $p < 0.00025$.

eTable 7: Average Marginal Component Effects (AMCE) for Patient Trust, full sample.

Attribute	Estimate	Std. Err	Pr(>|z|)	
AI data quality (white, male, wealthy)	-0.032	0.016	0.048	
AI data quality (representative)	0.236	0.016	0.000	*
AI performance (as general practitioner)	0.430	0.019	0.000	*
AI performance (as specialist)	0.512	0.020	0.000	*
AI performance (better than specialist)	0.675	0.020	0.000	*
Clinician present	0.341	0.014	0.000	*
FDA approved	0.208	0.013	0.000	*
Local hospital approved	0.120	0.013	0.000	*
Mayo approved	0.202	0.013	0.000	*

Number of Obs. = 36000
Number of Respondents = 3000
'*' 0.00025

eTable 7: Average Marginal Component Effects (AMCE) for Patient Trust, full sample. This table corresponds to Figure 2 (Panel b) in the main manuscript. We apply a Bonferroni corrected p-value of $p < 0.00025$.

eTable 8: Clustered two-tailed Wald test for a difference between the AMCE coefficients for AI performance (about the same as a general practitioner) and Clinician present, patient choice

Attribute	AMCE Coefficient	Std. Err. of difference	p-value
AI performance (as general practitioner)	0.191		
Clinician present	0.184		
Difference	0.007	0.009	0.462

eTable 9: Clustered two-tailed Wald test for a difference between the AMCE coefficients for FDA approved and Mayo approved, patient choice

Attribute	AMCE Coefficient	Std. Err. of difference	p-value
FDA approved	0.111		
Mayo approved	0.111		
Difference	-0.000	0.007	0.957

eTable 10: Clustered two-tailed Wald test for a difference between the AMCE coefficients for FDA approved and Local hospital approved, patient choice

Attribute	AMCE Coefficient	Std. Err. of difference	p-value
FDA approved	0.111		
Local hospital approved	0.078		
Difference	0.033	0.007	0.000

eTable 11: Clustered two-tailed Wald test for a difference between the AMCE coefficients for Mayo approved and Local hospital approved, patient choice

Attribute	AMCE Coefficient	Std. Err. of difference	p-value
Mayo approved	0.111		
Local hospital approved	0.078		
Difference	0.034	0.007	0.000

eTable 12: Clustered two-tailed Wald test for a difference between the AMCE coefficients for AI performance (about the same as a general practitioner) and AI performance (about the same as a specialist), patient choice

Attribute	AMCE Coefficient	Std. Err. of difference	p-value
AI performance (as general practitioner)	0.191		
AI performance (as specialist)	0.248		
Difference	-0.057	0.007	0.000

eTable 13: Clustered two-tailed Wald test for a difference between the AMCE coefficients for AI performance (about the same as a specialist) and AI performance (better than a specialist), patient choice

Attribute	AMCE Coefficient	Std. Err. of difference	p-value
AI performance (as specialist)	0.248		
AI performance (better than specialist)	0.325		
Difference	-0.077	0.007	0.000

eTable 14: Clustered two-tailed Wald test for a difference between the AMCE coefficients for AI performance (about the same as a general practitioner) and Clinician present, patient trust

Attribute	AMCE Coefficient	Std. Err. of difference	p-value
AI performance (as general practitioner)	0.431		
Clinician present	0.341		
Difference	0.090	0.024	0.000

eTable 15: Clustered two-tailed Wald test for a difference between the AMCE coefficients for FDA approved and Mayo approved, patient trust

Attribute	AMCE Coefficient	Std. Err. of difference	p-value
FDA approved	0.208		
Mayo approved	0.202		
Difference	0.006	0.018	0.756

eTable 16: Clustered two-tailed Wald test for a difference between the AMCE coefficients for FDA approved and Local hospital approved, patient trust

Attribute	AMCE Coefficient	Std. Err. of difference	p-value
FDA approved	0.208		
Local hospital approved	0.120		
Difference	0.087	0.018	0.000

eTable 17: Clustered two-tailed Wald test for a difference between the AMCE coefficients for Mayo approved and Local hospital approved, patient trust

Attribute	AMCE Coefficient	Std. Err. of difference	p-value
Mayo approved	0.202		
Local hospital approved	0.120		
Difference	0.082	0.018	0.000

eTable 18: Average Marginal Component Effects (AMCE) for Patient Choice, full sample with demographic controls

Attribute	Estimate	Std. Err	Pr(>|z|)	P-value (exact)	
AI data quality (white, male, wealthy)	-0.01	0.01	0.33	3.33E-01	
AI data quality (representative)	0.12	0.01	0.000	2.90E-80	*
AI performance (as general practitioner)	0.19	0.01	0.000	1.48E-160	*
AI performance (as specialist)	0.25	0.01	0.000	4.22E-266	*
AI performance (better than specialist)	0.33	0.01	0.000	0.00E+00	*
Clinician present	0.18	0.01	0.000	1.51E-250	*
FDA approved	0.11	0.01	0.000	1.18E-99	*
Local hospital approved	0.08	0.01	0.000	1.01E-53	*
Mayo approved	0.11	0.01	0.000	3.54E-103	*
Age					
18-25	(baseline)				
26-35	0.00	0.00	0.41	0.41	
36-45	0.00	0.00	0.55	0.55	
46-55	0.00	0.00	0.54	0.54	
56-65	-0.01	0.00	0.21	0.21	
66+	-0.01	0.00	0.12	0.12	
Sex					
Male	(baseline)				
Female	0.00	0.00	0.08	0.08	
Other	0.01	0.01	0.41	0.41	
Race					
White	(baseline)				
Black	0.00	0.00	0.72	0.72	
Hispanic	0.00	0.00	0.11	0.11	
Other	0.00	0.00	0.37	0.37	
Income					
< $50,000	(baseline)				
$50,000 - $99,999	0.00	0.00	0.99	0.99	
$100,000 - $149,999	0.00	0.00	0.94	0.94	
> $150,000	0.00	0.00	0.99	0.99	
Education					
High school or less	(baseline)				
Some college/2-yr degree	0.00	0.00	0.49	0.49	

4-yr/post-graduate degree	0.00	0.00	0.93	0.93

Number of Obs. = 35964
Number of Respondents = 2997
'*' 0.00025

eTable 18: Average Marginal Component Effects (AMCE) for Patient Choice with population covariates, full sample. Three observations were lost due to missing data; one due to missing data on race and two due to missing data on income. The exact p-values demonstrate that the results are robust to Bonferroni corrections for multiple hypothesis testing. The number of tests includes all the attribute-level combinations, gender subsets, weighted and non-weighted models, and balancing checks, resulting in the corrected p-value of 0.00025 (0.05/197).

eTable 19: Average Marginal Component Effects (AMCE) for Patient Trust, full sample with demographic controls

Attribute	Estimate	Std. Err	P-value	P-value (exact)	
AI data quality (white, male, wealthy)	-0.03	0.02	0.07	6.65E-02	
AI data quality (representative)	0.24	0.02	0.000	9.75E-51	*
AI performance (as general practitioner)	0.43	0.02	0.000	2.23E-116	*
AI performance (as specialist)	0.51	0.02	0.000	1.49E-149	*
AI performance (better than specialist)	0.67	0.02	0.000	2.54E-244	*
Clinician present	0.34	0.01	0.000	1.22E-135	*
FDA approved	0.21	0.01	0.000	3.24E-59	*
Local hospital approved	0.12	0.01	0.000	1.48E-20	*
Mayo approved	0.20	0.01	0.000	2.06E-58	*
Age					
18-25	(baseline)				
26-35	0.10	0.06	0.06	0.06	
36-45	0.13	0.06	0.02	0.02	
46-55	0.18	0.06	0.002	0.002	
56-65	0.22	0.06	0.000	0.0001	*
66+	0.12	0.06	0.04	0.04	
Sex					
Male	(baseline)				
Female	-0.14	0.03	0.000	3.00E-06	*
Other	-0.52	0.14	0.000	0.00016	*
Race					
White	(baseline)				
Black	0.21	0.05	0.000	5.50E-06	*
Hispanic	0.15	0.04	0.000	0.0001	*
Other	-0.03	0.05	0.64	0.64	
Income					
< $50,000	(baseline)				
$50,000 - $99,999	0.09	0.04	0.01	0.01	
$100,000 - $149,999	-0.05	0.05	0.33	0.33	
> $150,000	0.09	0.06	0.11	0.11	
Education					
High school or less	(baseline)				
Some college/2-yr degree	-0.11	0.04	0.003	0.003	

4-yr/post-graduate degree	-0.05	0.04	0.24	0.24

Number of Obs. = 35964
Number of Respondents = 2997
'*' 0.00025

eTable 19: Average Marginal Component Effects (AMCE) for Patient Trust with population covariates, full sample. Three observations were lost due to missing data; one due to missing data on race and two due to missing data on income. The exact p-values demonstrate that the results are robust to Bonferroni corrections for multiple hypothesis testing. The number of tests includes all the attribute-level combinations, gender subsets, weighted and non-weighted models, and balancing checks, resulting in the corrected p-value of 0.00025 (0.05/197).

eTable 20: Average Marginal Component Effects (AMCE) for Patient Choice, women

Attribute	Estimate	Std. Err	Pr(>|z|)	
AI data quality (white, male, wealthy)	-0.019	0.009	0.025	
AI data quality (representative)	0.116	0.008	0.000	*
AI performance (as general practitioner)	0.193	0.010	0.000	*
AI performance (as specialist)	0.252	0.010	0.000	*
AI performance (better than specialist)	0.323	0.010	0.000	*
Clinician present	0.192	0.007	0.000	*
FDA approved	0.118	0.007	0.000	*
Local hospital approved	0.080	0.007	0.000	*
Mayo approved	0.108	0.007	0.000	*

Number of Obs. = 19728
Number of Respondents = 1644
'*' 0.00025

eTable 21: Average Marginal Component Effects (AMCE) for Patient Choice, men

Attribute	Estimate	Std. Err	Pr(>|z|)	
AI data quality (white, male, wealthy)	0.012	0.009	0.199	
AI data quality (representative)	0.120	0.010	0.000	*
AI performance (as general practitioner)	0.187	0.010	0.000	*
AI performance (as specialist)	0.243	0.011	0.000	*
AI performance (better than specialist)	0.329	0.011	0.000	*
Clinician present	0.173	0.008	0.000	*
FDA approved	0.102	0.008	0.000	*
Local hospital approved	0.074	0.008	0.000	*
Mayo approved	0.116	0.008	0.000	*

Number of Obs. = 16008
Number of Respondents = 1334
'*' 0.00025

eTable 22: Paired two-tailed t-test for a difference in means between mentions of AI performance and mentions of a clinician

Concept mentioned[1]	Mean	Std. Err. of difference	95% CI
AI performance	0.257		
Clinician	0.227		
Difference	0.030	0.012	(0.007, 0.054)

[1] Concepts are coded as 1 if they are mentioned by the respondent and 0 if they are not. See above for coding rules.

eTable 23: Paired two-tailed t-test for a difference in means between mentions of a clinician and mentions of AI data quality

Concept mentioned	Mean	Std. Err. of difference	95% CI
Clinician	0.227		
AI data quality	0.135		
Difference	0.092	0.01	(0.072, 0.112)

eTable 24: Paired two-tailed t-test for a difference in means between mentions of AI data quality and mentions of the FDA.

Concept mentioned	Mean	Std. Err. of difference	95% CI
AI data quality	0.135		
FDA	0.118		
Difference	-0.017	0.008	(-0.034, 0.0001)

eTable 25: Paired two-tailed t-test for a difference in means between mentions of the FDA and mentions of the Mayo Clinic.

Concept mentioned	Mean	Std. Err. of difference	95% CI
FDA	0.118		
Mayo Clinic	0.096		
Difference	0.022	0.008	(0.007, 0.037)

eTable 26: Paired two-tailed t-test for a difference in means between mentions of the Mayo Clinic and mentions of ease or comfort.

Concept mentioned	Mean	Std. Err. of difference	95% CI
Mayo Clinic	0.096		
Ease or Comfort	0.057		
Difference	0.039	0.007	(0.026, 0.053)

eTable 27: Paired two-tailed t-test for a difference in means between mentions of ease and comfort and mentions of local hospital.

Concept mentioned	Mean	Std. Err. of difference	95% CI
Ease or Comfort	0.057		
Local Hospital	0.046		
Difference	0.011	0.006	(-0.0003, 0.022)

eTable 28: Paired two-tailed t-test for a difference in means between mentions of local hospital and mentions of trust.

Concept mentioned	Mean	Std. Err. of difference	95% CI
Local Hospital	0.046		
Trust	0.036		
Difference	0.01	0.005	(0.0003, 0.02)

eTable A29: Paired two-tailed t-test for a difference in means between mentions of trust and expressions of anti-AI sentiment

Concept mentioned	Mean	Std. Err. of difference	95% CI
Trust	0.036		
Anti-AI sentiment	0.036		
Difference	0.000	0.004	(-0.008, 0.008)

eFigures

eFigure 1: Patient Choice AMCEs, weighted full sample with 95% confidence intervals.

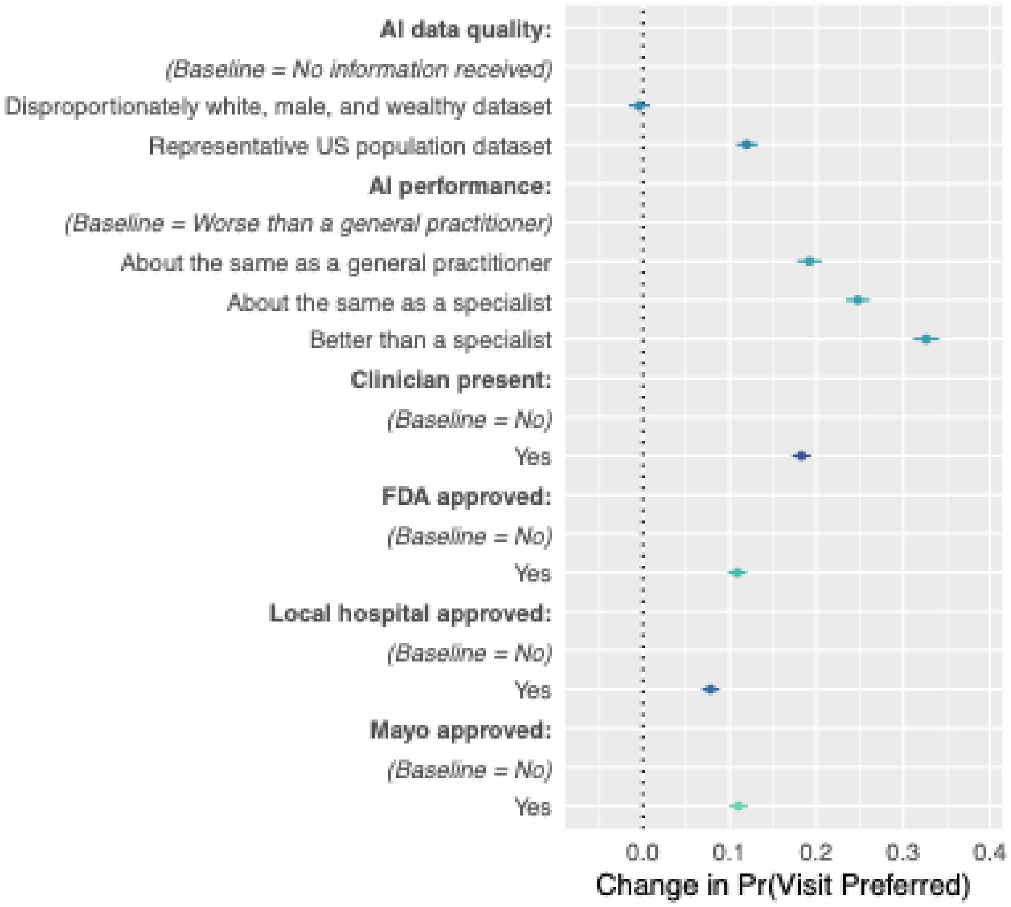

eFigure 1: Patient Choice AMCEs, weighted full sample with 95% confidence intervals. This figure shows the change in probability that a patient would prefer a hypothetical visit (in a binary choice where 1 indicates the visit was preferred) based on the different values for each characteristic. For each characteristic, multiple values are possible. The baseline (e.g., Clinician present: no) is not shown; the data points show the change from baseline presentation for other values of the characteristic (e.g., Clinician present: yes). Colors alternate by characteristic for ease of viewing. All changes are statistically significantly different from baseline at the p<0.00025 level except the biased training dataset (trained on a disproportionately white, male, and wealthy sample), which is not.

eFigure 2: Patient Choice AMCEs, women subset with 95% confidence intervals.

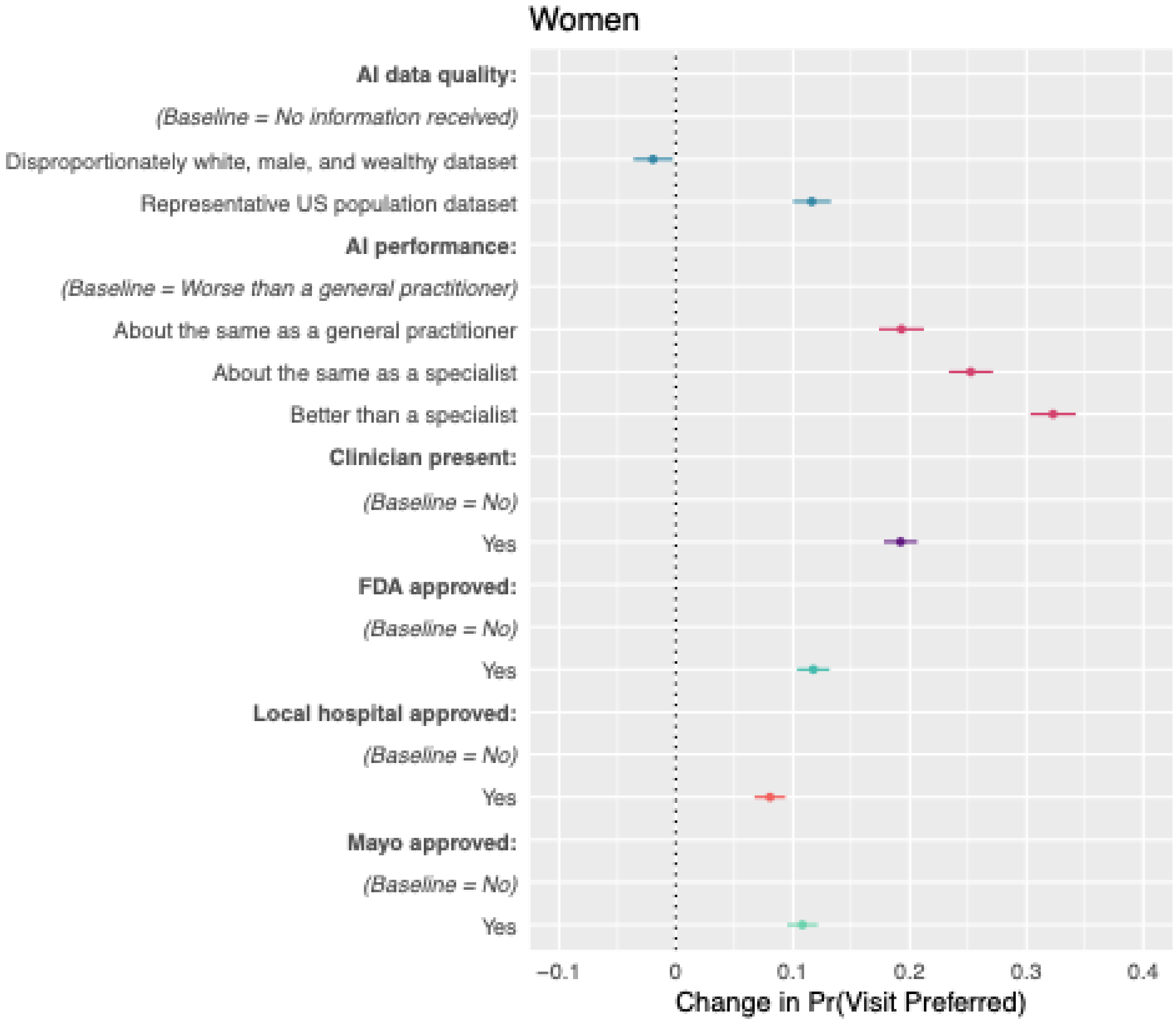

eFigure 2: Patient Choice AMCEs, women subset with 95% confidence intervals. This figure shows the change in probability that a patient would prefer a hypothetical visit (in a binary choice where 1 indicates the visit was preferred) based on the different values for each characteristic. For each characteristic, multiple values are possible. The baseline (e.g., Clinician present: no) is not shown; the data points show the change from baseline presentation for other values of the characteristic (e.g., Clinician present: yes). Colors alternate by characteristic for ease of viewing. All changes are statistically significantly different from baseline at the p<0.00025 level except the biased training dataset (trained on a disproportionately white, male, and wealthy sample), which is not.

eFigure 3: Patient Choice AMCEs, men subset with 95% confidence intervals.

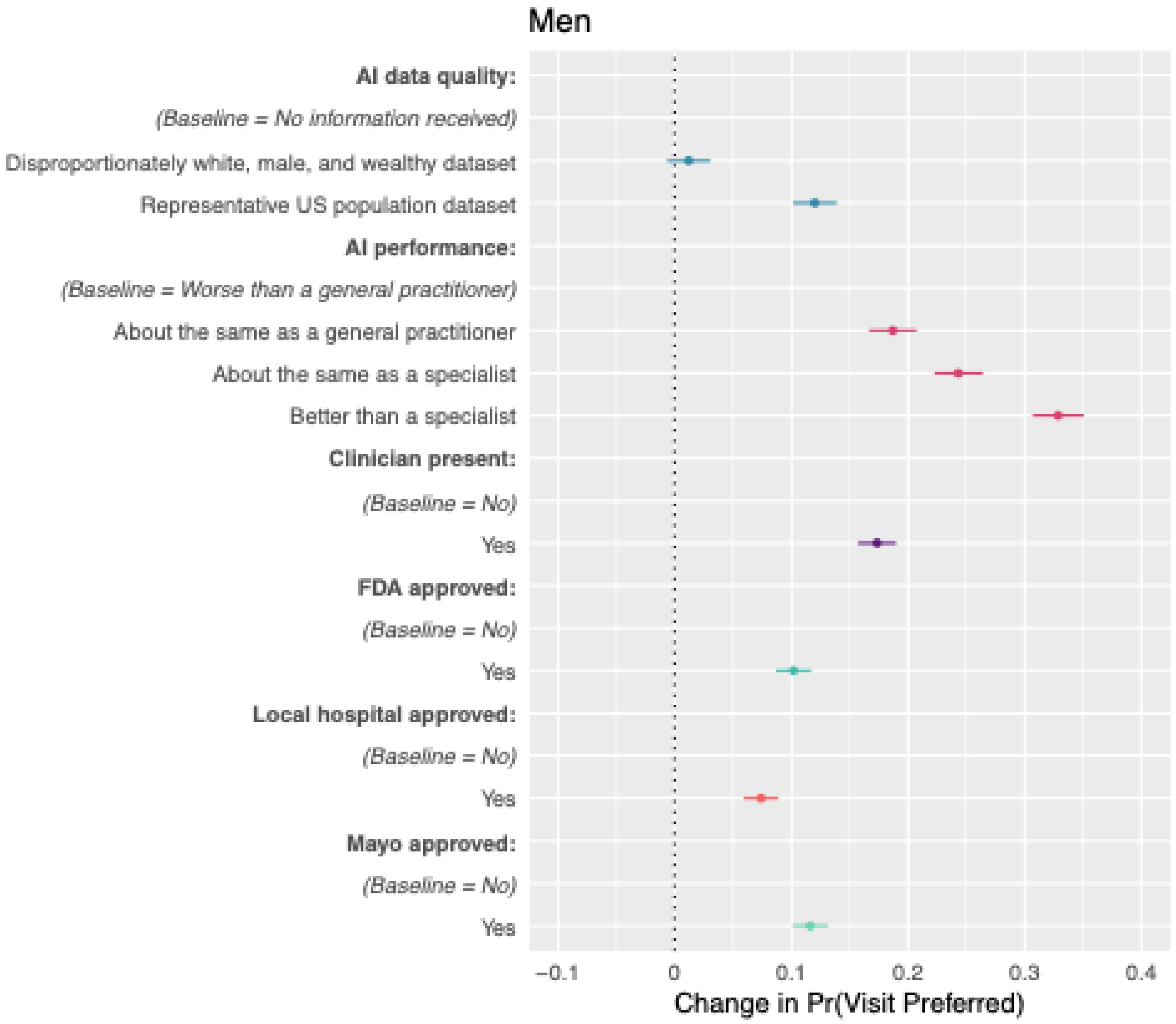

eFigure 3: Patient Choice AMCEs, men subset with 95% confidence intervals. This figure shows the change in probability that a patient would prefer a hypothetical visit (in a binary choice where 1 indicates the visit was preferred) based on the different values for each characteristic. For each characteristic, multiple values are possible. The baseline (e.g., Clinician present: no) is not shown; the data points show the change from baseline presentation for other values of the characteristic (e.g., Clinician present: yes). Colors alternate by characteristic for ease of viewing. All changes are statistically significantly different from baseline at the p<0.00025 level except the biased training dataset (trained on a disproportionately white, male, and wealthy sample), which is not.

eAppendix

Summary of results for Patient Trust

This summary refers to eTable 7.

All reported effects are significant at the p<0.001 level unless otherwise noted.

The respondents trust a hypothetical visit more when it includes a human in the loop (effect size of 0.341).

AI performance impacted patient trust more than clinician oversight. Patient trust was most enhanced by AI performance at or above specialist level (0.512 and 0.675, respectively). The effect for a medical AI that performs as well as a general practitioner (0.430) was significantly larger than the effect for clinician oversight (0.341, p<0.000[2]), which is the only substantive departure from the findings on patient choice, where effects for clinician presence and AI performing as well as a general practitioner were statistically indistinguishable.

Respondents trust AI trained on a representative US population dataset more than they trust AI about which they receive no training data information (0.236). They trust AI trained on a disproportionately white and male population significantly less than they trust AI about which they receive no training data information (0.032, p<0.048).

Respondents trust hypothetical visits more when they include a form of AI governance, compared to no governance. The effects of FDA approval (0.208) and Mayo clinic certification (0.202) are about the same size (p=0.756), while the effect of local hospital certification (0.120) is smaller (p<0.001 for differences between local and FDA or Mayo).

---

[2] See eTables 18-21 for full results from clustered two-tailed Wald tests that examine the differences between the coefficients.
