# Supplementary material for: Factors for Patient Trust and Acceptance of Medical Artificial Intelligence
Source: JAMA Netw Open. 2026 Mar 5;9(3):e260815. doi: 10.1001/jamanetworkopen.2026.0815 (PMC12964161; doi:10.1001/jamanetworkopen.2026.0815)
Supplement: Supplement 2. — Data Sharing Statement [file jamanetwopen-e260815-s002.pdf]

Data Sharing Statement

Bracic. Factors for Patient Trust and Acceptance of Medical Artificial Intelligence. *JAMA Netw Open*. Published March 05, 2026. doi:10.1001/jamanetworkopen.2026.0815

Data
Data available: No

Additional Information
Explanation for why data not available: The datasets generated during the current study are not publicly available due to privacy issues, but the corresponding author can be contacted with reasonable requests.
